# Differential hemodynamic and respiratory responses to right and left cervical vagal nerve stimulation in rats

**DOI:** 10.14814/phy2.13244

**Published:** 2017-04-11

**Authors:** Harald M. Stauss

**Affiliations:** ^1^ Department of Health and Human PhysiologyThe University of IowaIowa CityIowa

**Keywords:** Blood pressure, fiber recruitment, Heart rate, respiration rate, stimulation parameters

## Abstract

Neuromodulation through vagal nerve stimulation (VNS) is currently explored for a variety of clinical conditions. However, there are no established VNS parameters for animal models of human diseases, such as hypertension. Therefore, the aim of this study was to assess hemodynamic and respiratory responses to right‐ or left‐sided cervical VNS in a hypertensive rat model. Anesthetized stroke‐prone spontaneously hypertensive rats were instrumented for arterial blood pressure and heart rate monitoring and left‐ or right‐sided VNS. Cervical VNS was applied through bipolar coil electrodes. Stimulation parameters tested were 3 V and 6 V, 2 Hz to 20 Hz stimulation frequency, and 50 *μ*sec to 20 msec pulse duration. Each combination of stimulation parameters was applied twice with altered polarity, that is, anode and cathode in the cranial and caudal position. Respiration rate was derived from systolic blood pressure fluctuations. In general, cervical VNS caused bradycardia, hypotension, and tachypnea. These responses were more pronounced with left‐sided than with right‐sided VNS and depended on the stimulation voltage, stimulation frequency, and pulse duration, but not on the polarity of stimulation. Furthermore, the results suggest that at low stimulation frequencies (<5 Hz) and short pulse durations (<0.5 msec) primarily larger A‐fibers are activated, while at longer pulse durations (>0.5 msec) smaller B‐fibers are also recruited. In conclusion, in rats left‐sided cervical VNS causes greater cardio‐respiratory responses than right‐sided VNS and at lower stimulation frequencies (e.g., 5 Hz), longer pulse durations (>0.5 msec) seem to be required to consistently recruit B‐fibers in addition to A‐fibers.

## Introduction

Since the discovery of the anti‐inflammatory action of the parasympathetic nervous system (Tracey 2002), neuromodulation of vagal activity is increasingly explored as a potential treatment option for a variety of conditions associated with chronic inflammation, such as obesity (Pardo et al. 2007; Bodenlos et al. 2014), diabetes (Meyers et al. 2016), heart failure (Premchand et al. 2014; Zannad et al. 2015; Gold et al. 2016), hypertension (Chapleau et al. 2016), or diseases with an autoimmune component, such as lupus or rheumatoid arthritis (Das 2011). One important problem in the interpretation of such studies is that different investigators utilize different sites of vagal nerve stimulation (e.g., right vs. left cervical vagus nerve) and different stimulation parameters (e.g., voltage, frequency, pulse duration, polarity). Stimulating different branches of the vagus nerve (e.g., right‐ vs. left‐sided, cervical vs. subdiaphragmatic, etc.) may affect different target organs and different stimulation parameters may result in recruitment of different fiber types within the vagus nerve. Therefore, the physiologic response to vagal nerve stimulation is expected to depend on the site of stimulation and on the stimulation parameters chosen for a particular study. Furthermore, results obtained in one species may not necessarily apply to other species due to species differences in the neuroanatomy of the autonomic nervous system. Thus, the aim of this study was to explore the impact of different stimulation parameters, including voltage (which determines the stimulation current), frequency, pulse duration, and polarity (i.e., distal or proximal placement of anode and cathode of bipolar electrode) on hemodynamic and respiratory responses to right‐ or left‐sided cervical vagal nerve stimulation in rats, a species for which many human disease models exist and that has been used previously to investigate the effects of vagal nerve stimulation in pathologic conditions, such as obesity, diabetes, and hypertension (Bugajski et al. 2007; Chapleau et al. 2016; Meyers et al. 2016). While larger animal models may have advantages over rats with regard to translational potential and more genetically engineered mouse models than rat models are available, acute and chronic vagal nerve stimulation studies are technically more feasible in rats than in mice and vagal nerve stimulation studies in rats may serve as “proof of concept” studies before designing more costly and more elaborate studies in larger animals, such as dogs, sheep, pigs, or non‐human primates.

Therapeutic vagal nerve stimulation in patients with treatment‐refractory epilepsy or major depression usually targets the left cervical vagus nerve, because safety and efficacy of right vagal nerve stimulation has not been established (Cyberonics Physician's Manual, 2001; Navas et al. 2010). Safety issues of right‐sided cervical vagal nerve stimulation primarily concern unwanted cardiac effects, because in experiments in dogs right‐sided cervical vagal nerve stimulation evoked a significantly greater bradycardia than left‐sided stimulation (Ardell and Randall 1986). However, to the best of our knowledge no study directly compared the effects of right‐ versus left‐sided cervical vagal nerve stimulation on heart rate in rats.

The cervical vagal nerves are highly heterogeneous nerves and contain a much larger population of afferent (~80%) than efferent (~20%) nerve fibers (Ruffoli et al. 2011). Furthermore, the vagal nerves contain A‐, B‐, and C‐ fibers according to the classification by Erlanger and Gasser (1937) that is based on nerve conduction velocity. Vagal A‐fibers are the largest and myelinated fibers that carry afferent visceral information and efferent motor signals. B‐fibers are small and myelinated fibers that carry most of the efferent parasympathetic signals. Finally, vagal C‐fibers are small and unmyelinated and carry primarily afferent visceral information (Ruffoli et al. 2011). With regard to these different fiber types, it is important to note that they differ in their excitation thresholds (both current amplitude and pulse duration) at which they respond to electrical stimulation. A‐fibers have the lowest threshold followed by B‐ and C‐fibers. Thus, it is reasonable to assume that the fiber types predominately recruited by vagal nerve stimulation and, therefore, the physiologic response to vagal nerve stimulation depends on the stimulation parameters in such a way that with increasing stimulation intensities A‐fibers are recruited first, followed by B‐ and C‐fibers.

Another potentially important factor in nerve stimulation studies using bipolar electrodes is the polarity of the stimulation that is determined by the placement of the anode and cathode along the nerve. During application of an electrical stimulus, the nerve tissue adjacent to the cathode depolarizes and triggers an action potential, whereas the nerve tissue adjacent to the anode hyperpolarizes and may block the propagation of action potentials, a phenomenon referred to as anodal blocking (Brindley and Craggs 1980). Thus, placing the anode proximally from the cathode may block proximal propagation of action potentials and preferentially lead to efferent stimulation. On the other hand, placing the anode distally from the cathode may block distal propagation of action potentials and, thus, preferentially lead to afferent stimulation. Thus, the polarity of the stimulation may potentially alter the physiologic response to vagal nerve stimulation. However, it has been reported that anodal blocking of vagal nerve fibers in pigs requires relatively high stimulation currents in the range of 3.6–10.0 mA and pulse durations of 400–600 *μ*sec (Vuckovic et al. 2008).

This study was initially designed as a pilot study to identify stimulation parameters to be used in our recently published study on the effects of chronic vagal nerve stimulation on cardiovascular end‐organ damage in stroke‐prone spontaneously hypertensive rats (Chapleau et al. 2016). As such, the current study was not designed to investigate the physiology or pathophysiology of the parasympathetic nervous system or the role of the vagus nerve in general, but to provide an objective rationale for the selection of stimulation parameters and the site of stimulation (i.e., left vs. right cervical vagus nerve) in rats. The range of stimulation parameters tested was selected so that these parameters could technically be implemented in a small‐sized chronically implantable nerve stimulator for use in conscious rats. Furthermore, testing if the phenomenon of anodal blocking occurs with these specific stimulation parameters was important, because if anodal blocking occurred, this phenomenon could potentially be used for preferential efferent or afferent vagal nerve stimulation. The results presented in this manuscript may be useful for other investigators designing vagal nerve stimulation studies in rats.

## Methods

### Animals

The study was performed in male stroke‐prone spontaneously hypertensive rats at 15 ± 2 weeks of age (299 ± 21 g body weight). Rats were housed in clear plastic cages, and temperature and light periods (12‐h light‐dark cycle; light on between 6:00 am and 6:00 pm) were controlled. A standard rat chow and tap water were provided ad libitum. Experiments were approved by the Institutional Animal Care and Use Review Committee of the University of Iowa.

### Instrumentation

Rats were anesthetized using isoflurane (induction 5% in room air, maintenance during surgical instrumentation 1.5% to 2.5% in oxygen as needed) and kept under anesthesia throughout the experiment until euthanasia. Through an inguinal incision, a catheter was introduced into the left femoral artery and advanced into the abdominal aorta for blood pressure and heart rate monitoring. Then the right or left cervical vagus nerve was exposed through a midline neck incision and a bipolar stainless steel coil electrode was wrapped around the nerve for cervical vagal nerve stimulation. The nerve/electrode preparation was embedded in a silicon elastomer (Kwik‐Sil, World Precision Instrument, Inc., Sarasota, FL) for electrical insulation from the surrounding tissue.

### Experimental protocol

Following instrumentation, anesthesia was maintained using isoflurane in oxygen at a concentration between 1.2% and 1.5% as needed. Arterial blood pressure waveforms were recorded by connecting the femoral artery catheter to a pressure transducer (P23 ID, Gould‐Statham, Oxnard, CA) and amplifier (Series 4000, Gould, Inc., Cleveland, OH). The output of the amplifier was connected to an A/D‐converter (ADUSB4CH, Harald Stauss Scientific, Iowa City, IA) for data acquisition (500 Hz sampling rate) using the WinAD module of the freely available HemoLab software (http://www.haraldstauss.com/HaraldStaussScientific/hemolab). For vagal nerve stimulation, the bipolar cervical vagal nerve electrode was connected to an external stimulator (Stimulator SD9, Grass Instruments, Warwick, RI). Charge‐balanced (0.2 *μ*F serial capacitor) rectangular impulses were used for vagal nerve stimulation.

**Figure 1:**
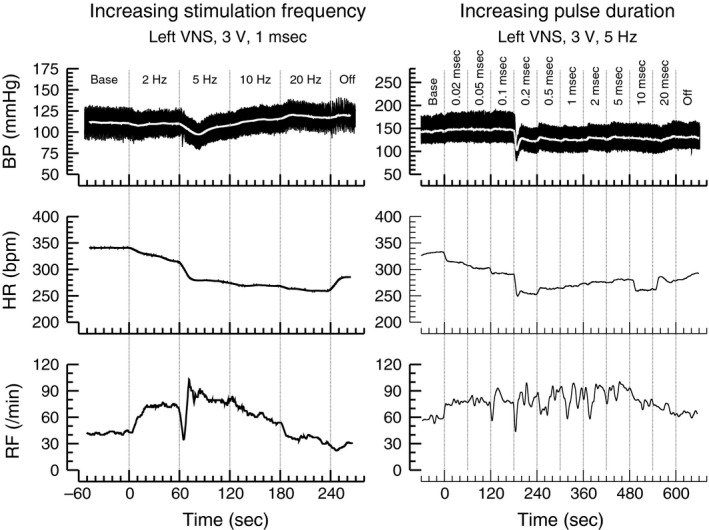
Original recordings of arterial blood pressure (BP, top), heart rate (HR, middle), and respiratory frequency (RF) obtained during the protocol with increasing stimulation frequencies ranging from 2 Hz to 20 Hz (left) and increasing pulse durations ranging from 0.02 msec to 20 msec (right). The lowest pulse duration of 0.02 msec was only tested in one more animals and, therefore, the pulse duration of 0.02 msec was not included in any statistical analyses.

In two stimulation protocols the effects of increasing pulse durations (0.05, 0.1, 0.2, 0.5, 1, 2, 5, 10, and 20 ms, *n* = 8) and increasing stimulation frequencies (2, 5, 10, and 20 Hz, *n* = 7) were investigated (Fig. 1). For both protocols, the effect of right versus left cervical vagal nerve stimulation and the effect of the polarity of the stimulation (active cathode and potentially blocking anode on either proximal or distal coil electrode) was investigated. For the protocol with increasing pulse durations the effect of the stimulation voltage (3 V vs. 6 V) was also investigated. Stimulation sequences with fixed stimulation voltage, polarity, and frequency (for increasing pulse duration protocol) or pulse duration (for increasing stimulation frequency protocol) were applied. Examples of such sequences with increasing pulse durations and stimulation frequencies are shown in Figure 1. After a stable baseline recording was obtained, the stimulation sequences were started with the shortest pulse duration (0.05 msec) or lowest stimulation frequency (2 Hz). Every minute the stimulation pulse duration or frequency was increased to the next higher value. Thus, the stimulation intensity gradually increased within each stimulation sequence. After all pulse durations or stimulation frequencies were applied, the stimulator was turned off and a new baseline was established for the next stimulation sequence using different stimulation parameters. The stimulation sequences were applied in randomized order. A minimum of 5 min without stimulation was allowed for recovery between different stimulation sequences. After this time, blood pressure, heart rate, and respiration rate had returned to baseline levels (see Tables 1 and 2).

**Table 1:** Baseline hemodynamic and respiratory parameters for the protocol with increasing pulse durations (0.05, 0.1, 0.2, 0.5, 1, 2, 5, 10, and 20 msec)

Cervical vagus	Left	Right	Left	Right	Left	Right
Voltage	3 V	3 V	3 V	3 V	6 V	6 V
Frequency	5 Hz	5 Hz	20 Hz	20 Hz	5 Hz	5 Hz
Number of rats	4	4	2	3	3	3
HR (bpm)	341 ± 5	317 ± 11	309 ± 12	303 ± 24	313 ± 7	311 ± 18
MAP (mmHg)	147 ± 8	134 ± 9	140 ± 20	128 ± 11	128 ± 5	112 ± 1
RF (min^−1^)	46 ± 6	41 ± 4	40 ± 1	46 ± 3	39 ± 7	34 ± 4

Baseline values for heart rate (HR), mean arterial blood pressure (MAP), and respiratory frequency (RF) before the beginning of cervical vagal nerve stimulation at different stimulation parameters (left or right cervical vagus nerve, voltage: 3 V or 6 V, stimulation frequency: 5 Hz or 20 Hz). Values are means±SEM. There were no significant differences between groups (independent measures one‐way analysis of variance).

**Table 2:** Baseline Hemodynamic and Respiratory Parameters for the Protocol with Increasing Stimulation Frequencies (2, 5, 10, and 20 Hz)

Cervical vagus	Left	Right	Left	Right
Pulse duration	0.1 msec	0.1 msec	1.0 msec	1.0 msec
Number of rats	3	4	3	4
HR (bpm)	327 ± 14	323 ± 19	330 ± 6	311 ± 18
MAP (mmHg)	126 ± 12	122 ± 8	121 ± 14	119 ± 7
RF (min^−1^)	36 ± 3	39 ± 5	36 ± 3	37 ± 4

Baseline values for heart rate (HR), mean arterial blood pressure (MAP), and respiratory frequency (RF) before the beginning of cervical vagal nerve stimulation at different stimulation parameters (left or right cervical vagus nerve, pulse duration: 0.1 ms or 1.0 ms). The stimulation voltage was 3 V. Values are means±SEM. There were no significant differences between groups (independent measures one‐way analysis of variance).

### Data analysis

For the protocol with increasing pulse durations, three sets of stimulation parameters (3V/5 Hz, 3V/20 Hz, 6V/5 Hz, see Table 1) and for the protocol with increasing stimulation frequencies two sets of stimulation parameters (3V/0.1 msec, 3V/1.0 msec, see Table 2) were tested for the right and left vagus nerve, respectively. The number of animals are provided in Tables 1 and 2. Each set of stimulation parameters was applied twice with alternating polarities. Thus, the analysis for the protocol with increasing pulse durations is based on a total number of 38 and the protocol with increasing stimulation frequencies on 28 stimulation sequences. For each stimulation sequence the arterial blood pressure waveforms were extracted in individual data files for the baseline preceding the stimulation sequence and for each of the nine applied pulse durations or nine applied stimulation frequencies for a total of 520 (380 from the pulse duration protocol and 140 from the stimulation frequency protocol) arterial blood pressure waveform files. Heart rate, systolic, mean, and diastolic blood pressure were extracted from all blood pressure waveform files using the Analyzer module of the HemoLab software (extracted on a beat‐by‐beat basis and then spline interpolated to an equidistant sampling rate of 20 Hz). Respiration frequencies were determined by identifying the frequency of the high frequency peak in the power spectrum of the 20 Hz equidistant systolic blood pressure signals using the fast Fourier transform (FFT) function of the Analyzer module of the HemoLab software. The baseline values for heart rate, mean blood pressure and respiration frequency preceding each stimulation sequence were subtracted from the values for each pulse duration or each stimulation frequency within the respective sequence of stimulation parameters. Multiple linear regression analysis and statistical analyses were performed from these delta values representing the changes in heart rate, mean blood pressure, and respiration rate in response to vagal nerve stimulation.

### Multiple linear regression analysis

This was performed on the data set consisting of heart rate, mean blood pressure, and respiration frequency responses to 454 applications of vagal nerve stimulation at different combinations of stimulation parameters (342 stimulations from the pulse width protocol and 112 stimulations from the frequency protocol). The independent parameters used for the multiple linear regression model included the stimulation voltage, the stimulation frequency, the pulse duration, the polarity of stimulation, and the site of vagal nerve stimulation (i.e., right or left cervical vagus nerve). The multiple linear regression analysis was performed using the free R software (Chambers 2008).

### Statistics

All data are presented as means ± SEM. Since the multiple linear regression analysis revealed that the stimulation polarity (cathode and anode on distal or proximal coil electrode) had no significant effect on the heart rate, blood pressure, or respiration rate responses (Table 3), stimulation sequences that only differed in the stimulation polarity were averaged for each animal for subsequent analyses. Statistical analysis was performed by two‐way Analysis of Variance (ANOVA) for one independent (left‐ vs. right‐sided vagal nerve stimulation) and one repeated measure (pulse duration or stimulation frequency). In case of significance in the two‐way ANOVA, post‐hoc Fisher tests were performed to identify significant differences between absolute values obtained during baseline conditions and vagal nerve stimulation at individual pulse durations or stimulation frequencies.

**Table 3:** Multiple linear regression analysis for the effect of different stimulation parameters on heart rate (HR), mean arterial blood pressure (MAP), and respiratory frequency (RF)

Parameter	ΔHR (bpm)	ΔMAP (mmHg)	ΔRF (min^−1^)
Intercept	−34.5 ± 6.4^a^	−19.7 ± 3.0^a^	19.0 ± 3.0^a^
Voltage (V)	3.75 ± 1.35^b^	2.08 ± 0.63^b^	2.14 ± 0.63^a^
Frequency (Hz)	−2.02 ± 0.26^a^	0.80 ± 0.12^a^	−0.58 ± 0.12^a^
Pulse duration (msec)	−1.26 ± 0.28^a^	0.09 ± 0.13 n.s.	−0.19 ± 0.13 n.s.
Proximal (1) or distal (‐1) cathode	0.24 ± 1.62 n.s.	−0.87 ± 0.75 n.s.	−0.20 ± 0.76 n.s.
Right (1) or left (‐1) VNS	9.35 ± 1.63^a^	1.87 ± 0.76^c^	−2.05 ± 0.76^b^

VNS, vagal nerve stimulation; n.s, not significant.

a *P* < 0.001.

b *P* < 0.01.

c *P* < 0.05.

## Results

In this study the impact of the stimulation voltage, frequency, pulse duration, and polarity on the effects of right‐ and left‐sided cervical vagal nerve stimulation on heart rate, mean arterial blood pressure and respiration rate was investigated. Examples of original recordings obtained during the two protocols, in which either the stimulation frequency or the stimulation pulse duration were varied are shown in Figure 1. Multiple linear regression analysis (Table 3) revealed that left‐sided cervical vagal nerve stimulation caused stronger bradycardic, hypotensive, and tachypneic effects than right‐sided vagal nerve stimulation. Furthermore, it indicated that the magnitude of these responses depends on the stimulation voltage and stimulation frequency. Interestingly, the pulse duration of the charge‐balanced rectangular impulses used for vagal nerve stimulation had a significant impact on the heart rate response, but not on the responses of mean blood pressure or respiration rate. Finally, the polarity of electrical stimulation (i.e., proximal or distal coil electrode connected as anode or cathode) did not have any significant impact on the responses of heart rate, mean blood pressure, or respiration rate, suggesting that phenomena such as anodal blocking did not occur.

### Impact of pulse duration

**Figure 2:**
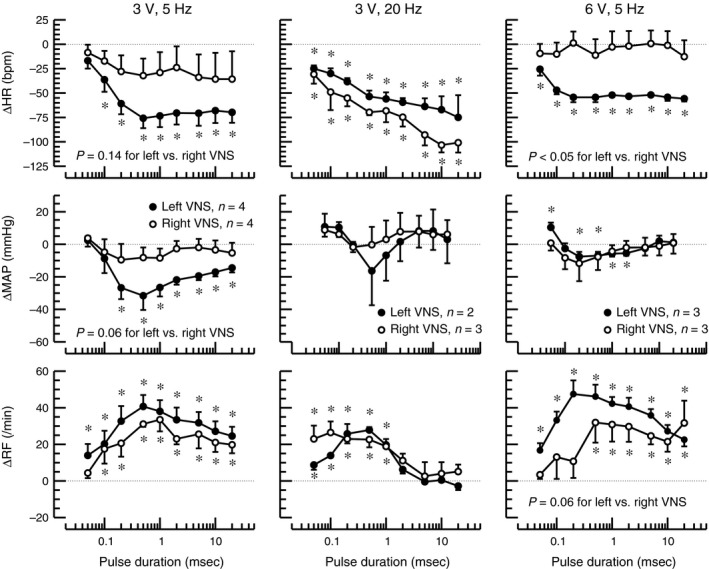
Changes (Δ) in heart rate (HR), mean arterial blood pressure (MAP), and respiratory frequency (RF) in response to left‐ (closed circles) or right‐sided (open circles) cervical vagal nerve stimulation using increasing pulse durations (x‐axes) of charge‐balanced rectangular impulses of 3 V or 6 V and 5 Hz or 20 Hz stimulation frequency. Absolute baseline values are provided in Table 1. *: P < 0.05 for difference of absolute values at the respective pulse duration and preceding baseline. P‐values for comparison of left versus right vagal nerve stimulation (VNS) are for group factor in 2‐way Analysis of Variance (ANOVA) with factors for right versus left VNS and for pulse duration. The number of animals for the three combinations of stimulation voltage and frequency are provided in the graphs for ΔMAP and also apply for ΔHR and ΔRF.

For left‐sided vagal nerve stimulation, lengthening the pulse duration of the charge‐balanced rectangular impulses generally increased the bradycardic effect (Fig. 2). At a stimulation frequency of 5 Hz, this bradycardic response reached a plateau at a pulse duration around 0.5 msec. However, at a stimulation frequency of 20 Hz and a voltage of 3 V, heart rate continued to fall as the pulse duration was increased up to 20 msec. Interestingly, right‐sided vagal nerve stimulation did barely affect heart rate at a stimulation frequency of 5 Hz (both 3 V and 6 V) but caused a bradycardic response, similar to that obtained with left‐sided vagal nerve stimulation, at a stimulation frequency of 20 Hz and a voltage of 3 V. Lengthening the pulse duration had only a minor effect on the blood pressure response to vagal nerve stimulation, although left sided vagal nerve stimulation at a combination of 3 V and 5 Hz caused a significant hypotensive response that was less pronounced at a voltage of 6 V and not observed with right‐sided vagal nerve stimulation. Respiration rate generally increased with vagal nerve stimulation and this tachypneic response peaked at a pulse duration around 0.5 msec. At the combination of 6 V and 5 Hz, pulse durations below 0.5 msec increased respiration rate only with left‐sided vagal nerve stimulation. Overall, these findings are consistent with the finding of the multiple linear regression analysis (Table 3), suggesting that left‐sided vagal nerve stimulation – in general – results in stronger heart rate, blood pressure, and respiratory responses than right‐sided vagal nerve stimulation.

### Impact of stimulation frequency

**Figure 3:**
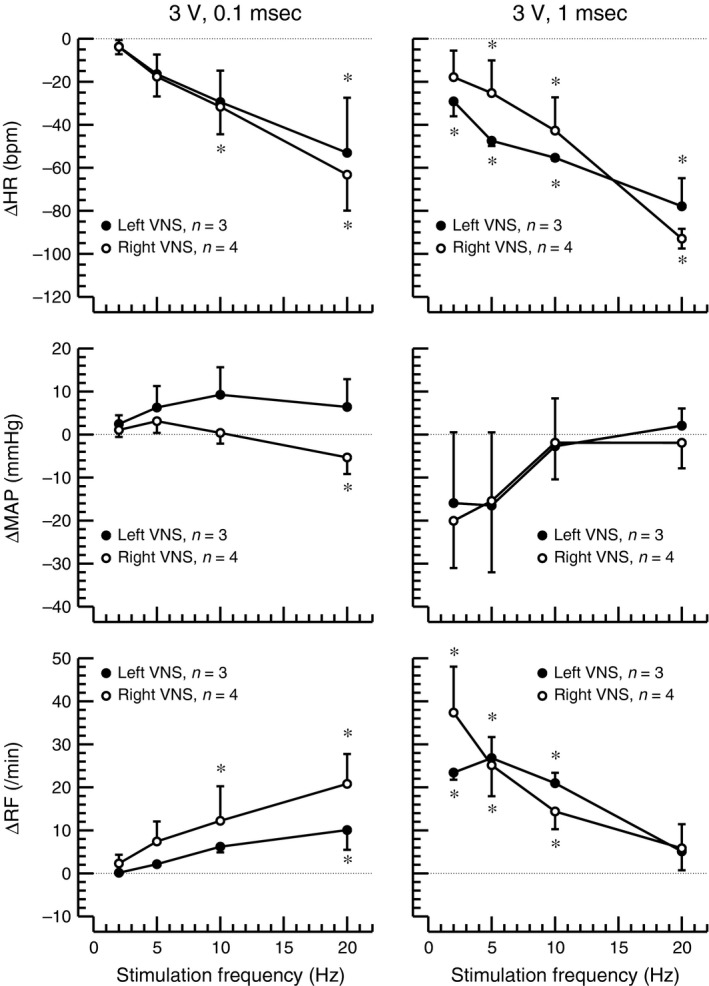
Changes (Δ) in heart rate (HR0, mean arterial blood pressure (MAP), and respiratory frequency (RF) in response to left‐ (closed circles, *n* = 3) or right‐sided (open circles, *n* = 4) cervical vagal nerve stimulation using increasing stimulation frequencies (x‐axes) of charge balanced rectangular impulses of 3 V and pulse durations of 0.1 msec (left) or 1.0 msec (right). Absolute baseline values are provided in Table 2. *: *P* < 0.05 for difference of absolute values at the respective stimulation frequency and preceding baseline. No significant differences were detected between right and left vagal nerve stimulation (VNS).

As the stimulation frequency was increased from 2 Hz to 5 Hz, 10 Hz, and 20 Hz, heart rate progressively decreased at both tested pulse durations (0.1 msec and 1.0 msec) (Fig. 3). This bradycardic response was similar for left‐ and right‐sided vagal nerve stimulation, although at a pulse duration of 1.0 msec, the lowest stimulation frequency tested (2 Hz) caused a significant bradycardia only for left‐sided vagal nerve stimulation. Increasing the stimulation frequency did not have a significant effect on mean arterial blood pressure except a small hypotensive response at the combination of 3 V, 0.1 msec, and 20 Hz for right‐sided vagal nerve stimulation. At the shorter pulse duration (0.1 msec), respiration rate increased as the stimulation frequency was increased. However, at the longer pulse duration (1.0 msec), even at the lowest stimulation frequency tested (2 Hz) a marked and significant increase in respiration rate was observed. Increasing the stimulation frequency above 2 Hz reduced this tachypneic effect observed at a pulse duration of 1.0 msec. No statistically significant differences in the respiratory responses were observed between right‐and left‐sided vagal nerve stimulation.

## Discussion

The most striking and potentially new finding of this study is that in rats left‐sided cervical vagal nerve stimulation caused stronger bradycardic, hypotensive, and tachypneic effects than right‐sided vagal nerve stimulation (Table 3). As expected, the magnitude of these responses to vagal nerve stimulation depended on the stimulation voltage (Table 3), pulse duration (Fig. 2), and stimulation frequency (Fig. 3). Finally, the polarity of the electrode did not affect the response to vagal nerve stimulation (Table 3), suggesting that anodal blocking does not occur with the range of stimulation parameters tested.

The finding that left‐sided vagal nerve stimulation caused stronger hemodynamic and respiratory effects than right‐sided vagal nerve stimulation is in contrast to findings in dogs that demonstrated greater bradycardia with right‐sided than left‐sided vagal nerve stimulation (Ardell and Randall 1986) and suggests a species difference between dogs and rats that may be partly due to the different anatomy in rats and dogs. For example, the aortic depressor nerve is a separate nerve branch in rats (Sato et al. 1999) but contained within the vagus nerve sheath in larger species, such as dogs (Walgenbach et al. 1981) and humans (Guyton 1991). Thus, VNS in larger species, including humans, must consider potential implications of aortic baroreceptor/chemoreceptor stimulation that is not occurring with VNS in rats. Such species differences may potentially have important implications, because the assumption that left‐sided vagal nerve stimulation is safer than right‐sided vagal nerve stimulation in humans (Cyberonics Physician's Manual, 2001) is largely based on studies performed in species other than humans. There are a few clinical case reports from patients in whom right‐sided vagal nerve stimulation was performed after complications with previous left‐sided vagal nerve stimulation, such as infections (McGregor et al. 2005; Spuck et al. 2008; Navas et al. 2010). The number of cases in these studies is rather low and does not allow for a definite answer to the question whether cardiac or respiratory side effects are more prevalent with left‐ or right‐sided vagal nerve stimulation in humans. But overall, these few case reports do not point toward an alarmingly high prevalence of cardiac or respiratory side effects with right‐sided vagal nerve stimulation but rather suggest that right‐sided vagal nerve stimulation may be as safe as left‐sided vagal nerve stimulation in humans. Interestingly, the differential heart rate response to left‐ versus right‐sided vagal nerve stimulation in our study was only apparent at a stimulation frequency of 5 Hz (Fig. 2, left and right columns). At a stimulation frequency of 20 Hz left‐and right‐sided vagal nerve stimulation had similar heart rate effects (Fig. 3, middle column). Thus, to minimize cardiac side effects of vagal nerve stimulation in rats a stimulation frequency of 5 Hz applied to the right cervical vagus nerve may be selected.

Recruitment of different fiber types within the vagus nerve depends on the threshold stimulus that is determined by the total charge delivered to the nerve fiber. This total charge depends on the stimulation current (which in our setup depended on the stimulation voltage), the pulse duration of the rectangular charge‐balanced impulses used for vagal nerve stimulation and the rate at which these pulses are applied, which is the stimulation frequency. In general, higher stimulation currents/voltages, longer pulse durations, and/or higher stimulation frequencies are needed to activate smaller nerve fibers. The heart rate response to vagal nerve stimulation is primarily mediated by B‐fibers that carry most of the efferent parasympathetic signals. At a voltage of 3 V and a stimulation frequency of 5 Hz, the full heart rate response required a pulse duration of 0.5 msec (Fig. 2, left), suggesting that a pulse duration of 0.5 msec is required to recruit B‐fibers. However, at shorter pulse durations the threshold for B‐fibers may also be reached by increasing the stimulation frequency or voltage, because a maximum heart rate response was achieved with a pulse duration of 0.2 msec at 5 Hz and 6 V (Fig. 2, right) and a pronounced heart rate response was also observed at a pulse duration of 0.1 msec if the stimulation frequency was raised to 20 Hz (Fig. 3). Interestingly, for the respiratory response, a bi‐phasic response was observed in response to increasing pulse durations. Up to a pulse duration of 0.5 msec, respiration rate increased with increasing pulse widths but then decreased as the pulse duration was lengthened beyond 0.5 msec (Fig. 2). The question if recruitment of different fiber types at pulse durations below and above 0.5 msec is responsible for this biphasic response cannot be answered directly from the data obtained in this study, but this possibility certainly exists.

With bipolar nerve stimulation as performed in this study, action potentials are triggered at the cathode, while the anode exerts a hyperpolarizing effect on the neurons. This phenomenon can potentially lead to a phenomenon referred to as anodal blocking (Brindley and Craggs 1980). Switching the polarity of stimulation had no systematic effect on the heart rate, mean blood pressure, or respiratory response to vagal nerve stimulation (Table 3). Thus, anodal blocking does not seem to occur at the range of stimulation parameters tested in this study.

The data presented in this manuscript may guide other investigators in the selection of stimulation parameters for vagal nerve stimulation studies in rats. For example, the data obtained in this study were used to determine the stimulation parameters in our recently published study on the effects of chronic vagal nerve stimulation on endothelial dysfunction and aortic stiffening in stroke‐prone spontaneously hypertensive rats (Chapleau et al. 2016). To minimize cardiorespiratory side effects, we opted for right‐sided rather than left‐sided vagal nerve stimulation. Because we hypothesized that an anti‐inflammatory effect mediated by the parasympathetic nervous system is involved in the vascular effects of chronic vagal nerve stimulation, we wanted to recruit B‐fibers that carry most of the efferent parasympathetic signals. Thus, we selected a rather long pulse duration of 1 msec. Because the differential response of left‐ versus right‐sided vagal nerve stimulation was not apparent at 20 Hz (Fig. 2) and because we wanted to avoid the stronger cardiorespiratory responses of left‐sided vagal nerve stimulation we decided to use a stimulation frequency of 5 Hz. Because the cardiorespiratory responses of right‐sided vagal nerve stimulation at 5 Hz and 1 msec were similar at 3 V and 6 V (Fig. 2), we decided to use 3 V instead of 6 V, which had the additional advantage that the stimulator could be manufactured with a smaller and less heavy battery.

### Limitations

This study was originally designed as a pilot study to identify stimulation parameters for a larger study (Chapleau et al. 2016). As such, a limited number of animals were used which resulted in a somewhat low statistical power if the two protocols (increasing pulse durations and increasing stimulation frequencies) are considered individually. This limitation was particularly apparent in the protocol with increasing stimulation frequencies were the different responses to right and left vagal nerve stimulation were not as apparent as for the protocol with increasing pulse durations. To overcome this limitation, we combined all data from both protocols in a single multiple linear regression analysis (Table 3). This analysis is based on 448 degrees of freedom and resulted in an overall multiple R value of 0.48 and an overall *P* value of 2.2*10^−16^. Thus, the differential responses of heart rate, mean blood pressure, and respiration frequency to right versus left vagal nerve stimulation identified in this study appears to be a highly reliable finding. Another potential limitation is that this study was performed in stroke‐prone spontaneously hypertensive rats. Different responses to vagal nerve stimulation may potentially be obtained in other strains of rats (e.g., normotensive strains).

In conclusion, in rats left‐sided cervical vagal nerve stimulation appears to cause stronger cardiorespiratory responses than right‐sided cervical vagal nerve stimulation. The results of this study suggest that at low stimulation frequencies (5 Hz) and short pulse durations (<0.5 msec) primarily A‐fibers are activated. Increasing the pulse duration (>0.5 msec) may additionally recruit B‐ (and possibly C) fibers. Finally, anodal blocking does not seem to occur at the stimulation parameters tested in this study.

## Conflict of Interest

Harald Stauss is founder of Harald Stauss Scientific, a company that markets implantable vagal nerve stimulators for rodents.
